# A pedicled latissimus dorsi myocutaneous flap for early reconstruction of cervicocranial necrotising fasciitis

**DOI:** 10.4103/0970-0358.73470

**Published:** 2010

**Authors:** KS Alexander, GS Lawton, AHF MacQuillan

**Affiliations:** Department of Plastic and Reconstructive Surgery, Royal Free Hospital, Pond Street, London, NW3 2QG, UK

Sir,

Craniocervical necrotising fasciitis (CCNF) accounts for only 5% of all NF cases.[1] Poorer outcomes are seen in the elderly, patients with concomitant disease, the immuno-compromised and those with streptococcal toxic shock syndrome (STSS).[2] The reconstruction of the resulting defect can be complex as a result of exposure to vital structures. We present a case of a 69-year-old lady with multiple comorbidities – chronic renal failure (requiring continuous ambulatory peritoneal dialysis), hyperparathyroidism, type II diabetes mellitus and bipolar affective disorder – who developed CCNF with resulting exposed carotid sheath and brachial plexus.

This patient was admitted under the renal physicians for confusion and ataxia, with a three-week history of a sore throat. Forty-eight hours after admission, she developed a fluctuant area over her left supraclavicular fossa with overlying cellulitis and a temperature of 38.8°C. Seventy-two hours post-admission, necrosis of the overlying skin prompted a plastic surgery referral. The diagnosis of CCNF was made and an emergency surgery was planned.

**Figure 1:**
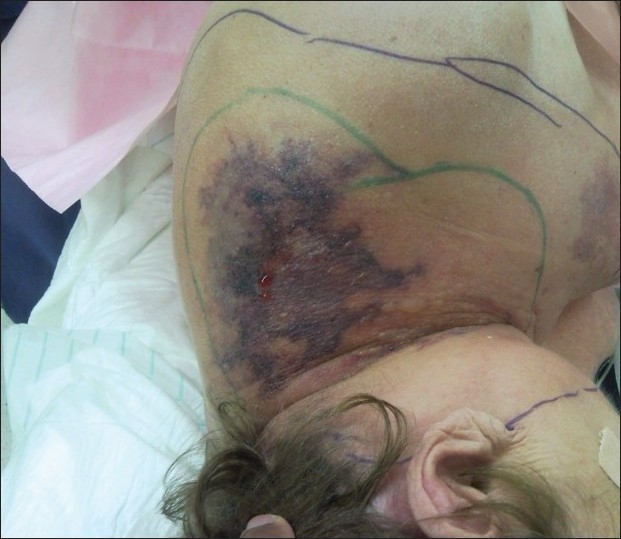
Pre-debridement

**Figure 2:**
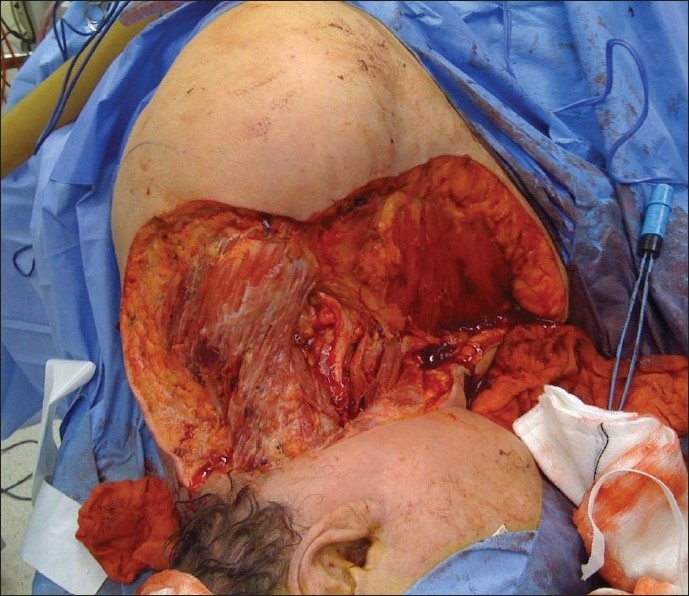
Post-debridement

The intra-operative findings were of necrotic fascia and subcutaneous fat with frank pus in the tissue planes [Figure 1], extending into the left anterior and posterior triangles of the neck and also the superior halves of trapezius and pectoralis major. All necrotic tissue was debrided, including an infarcted accessory nerve. The carotid sheath and a partially denuded brachial plexus were left exposed by the debridement [Figure 2]. The omohyoid was released from its insertion, and turned back to provide temporary cover of the carotid sheath. This could be done without devascularising the muscle, as its blood supply enters the superior belly.[3] Blood culture showed the growth of group A beta haemolytic streptococcus, and anaerobic tissue culture grew Serratia marcescens. She was treated with clindamycin, benzyl penicillin and ciprofloxacin as per the sensitivity studies.

On day 2, she developed some necrotic patches on her left forearm, which were debrided. Samples did not grow any organisms and these were presumed to be secondary to pressure areas from being in a right lateral position in her initial debridement.

**Figure 3:**
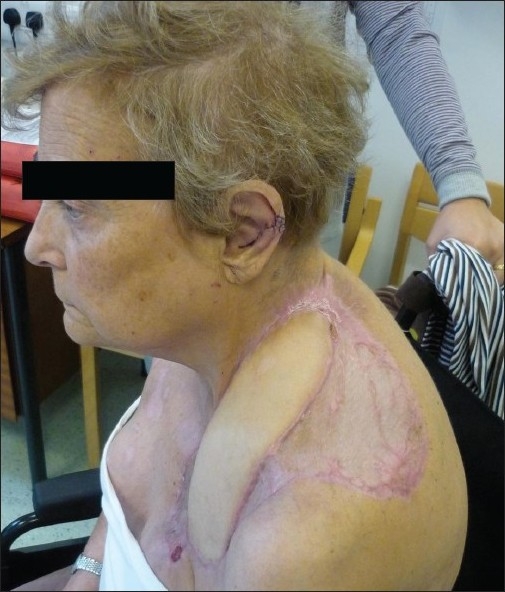
Post-reconstruction

On day 4, the defect was reconstructed with a pedicled myocutaneous latissimus dorsi flap (a muscle-only latissimus dorsi flap with a skin graft was not chosen, as secondary contraction of the skin graft would have resulted in a flexion contracture of the neck). The insertion of latissimus dorsi was released to facilitate transposition of the flap to cover the defect, with the flap being tunnelled between the two heads of pectoralis major to gain access anteriorly. Peripheral areas not covered by the skin paddle were reconstructed using split thickness skin grafts. A pedicled pectoralis major flap was deemed not suitable, as the thoracoacromial vessels were thought likely to have been damaged by disease process. She did not require any further operative interventions. On discharge her functional deficit was confined to limited left shoulder abduction at 90° [Figure 3].

Whilst we accept that skin grafts could have been used to cover the defect, this would have almost certainly committed her to numerous revision surgeries to correct the inevitable contractures. This case highlights the fact that severely immunocompromised patients can cope with pedicled flap reconstruction in the acute setting of necrotising fasciitis to facilitate coverage of exposed vital structures.
